# Increased EEG coherence in long‐distance and short‐distance connectivity in children with autism spectrum disorders

**DOI:** 10.1002/brb3.1796

**Published:** 2020-08-19

**Authors:** Jia Wang, Xiaomin Wang, Xuelai Wang, Huiying Zhang, Yong Zhou, Lei Chen, Yutong Li, Lijie Wu

**Affiliations:** ^1^ Department of Children’s and Adolescent Health Public Health College of Harbin Medical University Harbin China; ^2^ Department of Developmental and Behavioral Pediatrics Shanghai Children's Medical Center Shanghai Jiao Tong University School of Medicine Shanghai China; ^3^ Heilongjiang Province Center for Disease Control and Prevention Harbin China

**Keywords:** autism spectrum disorder, coherence, electroencephalogram, resting state

## Abstract

**Introduction:**

Autism spectrum disorder (ASD) is a complex and prevalent neurodevelopmental disorder characterized by deficits in social communication and social interaction as well as repetitive behaviors. Alterations in function connectivity are widely recognized in recent electroencephalogram (EEG) studies. However, most studies have not reached consistent conclusions, which could be due to the developmental nature and the heterogeneity of ASD.

**Methods:**

Here, EEG coherence analysis was used in a cohort of children with ASD (*n* = 13) and matched typically developing controls (TD, *n* = 15) to examine the functional connectivity characteristics in long‐distance and short‐distance electrode pairs. Subsequently, we explore the association between the connectivity strength of coherence and symptom severity in children with ASD.

**Results:**

Compared with TD group, individuals with ASD showed increased coherence in short‐distance electrode pairs in the right temporal–parietal region (delta, alpha, beta bands), left temporal–parietal region (all frequency bands), occipital region (theta, alpha, beta bands), right central–parietal region (delta, alpha, beta bands), and the prefrontal region (only beta band). In the long‐distance coherence analysis, the ASD group showed increased coherence in bilateral frontal region, temporal region, parietal region, and frontal–occipital region in alpha and beta bands. The strength of such connections was associated with symptom severity.

**Discussion:**

Our study indicates that abnormal connectivity patterns in neuroelectrophysiology may be of critical importance to acknowledge the underlying brain mechanism.

## Graphical abstract

Alterations in function connectivity are widely recognized in recent electroencephalogram (EEG) studies. Our study indicates that discovery of atypical brain coherence in ASD young children may be of a critical importance to acknowledge the underlying brain mechanism.

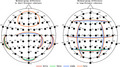

## 1 INTRODUCTION

Autism spectrum disorder (ASD) is a pervasive neurodevelopmental disorder with deficits in social communication and social interaction as well as restricted, repetitive pattern of behaviors, interests, and/or activities (American Psychiatric Association, 2013). According to the most recent survey, the prevalence of ASD among children aged 8 years reached 1/59 (Baio et al., 2018), which makes ASD a severe burden for whole family and society. Multiple causes are implicated in ASD, and recent imaging evidence indicates the presence of atypical brain structure and brain function during brain development. The research using structural magnetic resonance imaging (sMRI) found increased surface area for all cortical regions (Hazlett et al., 2011) and increased head circumference in the ASD group (Courchesne, 2002). Atypical brain anatomy and neurodevelopment significantly related to functional changes. The hypothesis that ASD is characterized by disrupted functional connectivity has been reliably supported by functional magnetic resonance imaging (fMRI) studies (Rane et al., 2015). Some fMRI studies observed disproportionate activation of ASD in the amygdala (Monk et al., 2010), cingulate gyrus (Shafritz, Dichter, Baranek, & Belger, 2008), fusiform gyrus (Pierce & Redcay, 2008), and other brain regions in the task state. Our previous resting state fMRI study showed alterations in functional connectivity in ASD boys aged 3–7 years, ASD children displayed significantly stronger functional connectivity between left postcentral gyrus and right angular gyrus, superior parietal gyrus and superior occipital gyrus, which may serve as important indicators of disease severity (Jia et al., 2017). According to previous MRI studies (Jia et al., 2017; Monk et al., 2010; Shafritz et al., 2008), ASD may be typically associated with widely distributed alterations of brain anatomy and functional connectivity with high spatial resolution. Despite a number of serious attempts, there are as yet no universally established centralized criteria that related to characterize ASD. This undoubtedly reflects the complexity of the underlying neural mechanism in ASD.

Electroencephalogram (EEG), which is known for its canonical and noninvasive advantages, is specialized in recording brain electrical activity generated by neurons and reveals brain connective characters. EEG includes delta (0.5–3.5 Hz), theta (3.5–7.5 Hz), alpha (7.5–12.5 Hz), beta (12.5–30 Hz), and gamma (30–100 Hz) frequency bands (Buzsáki & Draguhn, 2004). EEG power study pointed that 3‐month infants at high family risk of ASD show reduced frontal high‐alpha power (Levin, Varcin, O’Leary, Tager‐Flusberg, & Nelson, 2017). Resting state EEG studies in children and adults with ASD found widespread increased delta band power, including frontal lobe (Pop‐Jordanova, et al, 2010; Stroganova, et al, 2007), parietal lobe, and right temporal lobe (Chan et al, 2007). However, the largest reductions in absolute delta power in children with ASD are in the left frontal and posterior regions (Coben, Clarke, Hudspeth, & Barry, 2008). The difference of age may be the reason why the results of power spectrum of individual ASD are inconsistent, so the characteristics of resting power spectrum related to ASD still need to be further explored. EEG coherence, a main method of EEG functional connectivity analysis, has been used to explore brain connectivity pattern and how different brain areas communicate with each other based on high temporal resolution. Two EEG coherence studies reported under‐ and over‐connectivity in different frequency bands in ASD patients (Courchesne, Campbell, & Solso, 2011; Murias, Webb, Greenson, & Dawson, 2007). The prevailing hypothesis in imaging studies is that brain connectivity of ASD is characterized by reduced long‐range functional connectivity and increased local functional connectivity (Barttfeld et al., 2011; Belmonte et al., 2004; Minshew & Williams, 2007; Rubenstein & Merzenich, 2003). Enhanced local coherence has been found over lateral‐frontal region in the delta band (Barttfeld et al., 2011), over left frontal and temporal regions in the theta band (Murias et al., 2007). By contrast, both intrahemispheric and interhemispheric reduced local coherence in all brain regions have been reported in delta and theta bands (Coben, Clarke, et al., 2008), while reduced local coherence over mid‐frontal region has been reported in the delta (Barttfeld et al., 2011) and alpha bands (Murias et al., 2007). However, EEG coherence demonstrated increased long distance for individuals with ASD (Duffy & Als, 2012). Machado et al. (2015) found that significantly higher intrahemispheric long‐range coherence in the left hemisphere in ASD children, supporting the hypothesis of over functional connectivity in ASD. Differently, Carson et al. supported that children with ASD displayed reduced long‐distance coherence at the alpha frequency during resting state (Carson, Salowitz, Scheidt, Dolan, & Van Hecke, 2014). Coben et al. elaborated that children with ASD showed decrease interhemispherical and intrahemispherical coherence in delta and theta frequency bands (Coben, Clarke, et al., 2008). Although the underlying understanding of the essence of functional connectivity in ASD is incomplete, current findings suggest that abnormal structural and functional connectivity patterns may be the potential neural mechanisms of typical cognitive and behavioral impairments in ASD.

Autism spectrum disorder is an early‐onset disorder. Hyperconnectivity was prominent over frontal and central areas; the degree of hyperconnectivity at 14 months strongly correlated with the severity of restricted and repetitive behaviors in participants with ASD at 3 years in the alpha band (Orekhova et al., 2014). Over the past years, EEG coherence has been associated with clinical behavioral disorders in patients with ASD. Increasing evidence indicates that the primary reason of abnormal cognition and behavior in children with ASD lies in the defect of executive function (Gilotty, Kenworthy, Sirian, Black, & Wagner, 2002). Some researchers found that both patients with executive dysfunction and high functional ASD patients showed higher coherence on theta frequency band in resting state (Babiloni et al., 2004; Ford, Mathalon, Whitfield, Faustman, & Roth, 2002). Han YM combined clinical severities with coherence and suggested that the degree of executive dysfunction in patients with ASD may be closely related to their neural connection disorders (Han & Chan, 2017).

Autism spectrum disorder is a dynamic disorder with complex changes over time from childhood into adulthood (Lange et al., 2015). The hypothesis of early brain overgrowth in children with ASD among aged 2–4 years is one of the most prominent theories (Courchesne et al., 2001; Sparks et al., 2002). To our knowledge, many literature on EEG coherence have focused on autistic adolescents and adults, but only a few EEG studies have concerned with children with ASD. In this study, we used resting state EEG coherence to reveal functional connectivity characters in children with ASD. We hypothesized that long‐distance and short‐distance coherence would be atypical in ASD, and these alterations in functional connectivity may be associated with symptom severity. Our findings provide electrophysiological evidence to further understand the characteristics of brain connectivity patterns in children with ASD.

## 2 MATERIALS AND METHODS

### 2.1 Participants

Totally, 27 children with ASD were recruited from the Children Development and Behavioral Research Center of Harbin Medical University, and 41 TDs were recruited from the local kindergartens. None of them had genetic, neurological, or other psychiatric disorders, and none of the children were taking psychotropic medications. Written informed consent was obtained from the guardians of each subject prior to examination. This study protocol was approved by the ethics review committee of Harbin Medical University (Approval number: 2013004).

### 2.2 Diagnosis and clinical assessment

The ASD diagnosis was based on DSM‐5(American Psychiatric Association, 2013), combined with the Autism Diagnostic Interview‐Revised (ADI‐R; Lord, Rutter, & Couteur, 1994) and Autism Diagnostic Observation Schedule (ADOS) (Lord et al., 2000). In addition, the overall intelligence level of all children was estimated by Peabody Picture Vocabulary Test (PPVT) (Dunn, Dunn, & Arribas, 2006). The Social Responsiveness Scale (SRS) was also applied to estimate their ability to engage in emotionally appropriate social interactions (Constantino, 2002). The Autism Spectrum Quotient Children's Version (AQ‐child; Baron‐cohen, Wheelwright, Skinner, Martin, & Clubley, 2001) is suitable for 4–11 years old children with autism screening. The items in this scale were divided into 5 dimensions, including social skills, attention switching, attention to detail, communication, and imagination. Each item was scored with 0–3 points and 4 grades. The total score of the scale was 0–150. The higher the total score, the more serious the autistic symptoms were.

### 2.3 EEG data acquisition

The experiment was carried out in a quiet and well‐lit electrophysiological room. All subjects were awake, relaxed, kept stationary, and sat comfortably in their chairs. Due to children with ASD had abnormal sensory behavior, we played cartoon clips to children in the process of wearing electrode cap and experiment preparation to stabilize their mood and improve coordination. The resting state EEG data were collected using 64‐channel of the event‐related potential recording system and PyCorder software (Brain Products) with a sampling rate of 500 Hz. All subjects wore 64‐channel acticap electrode cap (Brain Products). The electrodes were placed in accordance with the international 10–20 system (Klem, Luders, Jasper, & Elger, 1999), and the ground electrode was located between the Fp1 and Fp2 electrodes. We used bilateral mastoid electrodes as the reference electrodes and recorded vertical electrooculography (VEOG) and the horizontal electrooculography (HEOG) to monitor eye blink and eye movement signals. Impedance was set at less than 5 kΩ. All the children were asked to look at the green "+" in the middle of the black screen as long as possible during the EEG data acquisition process. A total of 2.5 min open‐eyes EEG data were obtained for each participant.

### 2.4 EEG data preprocessing

Because the children in the study were young, although we adopted a series of methods to ensure the data acquisition process proceeded smoothly, some children still had poor coordination, such as limb movement during the experiment, which caused large artifacts in the EEG signals. In order to ensure the reliability of the results of the study, these subjects were excluded from this study. Finally, this study included 13 autistic children and 15 sex‐, age‐, handedness‐matched healthy children (Table 1). Data preprocessing was conducted by the Brain Vision Analyzer software (Brain Products, GmbH). The steps included the following: (a) data filtering: Band‐pass filters were set at 0.01–30 Hz, (b) artifact rejection: Independent component analysis (ICA) was applied to the EEG signals, and the components responsible for the eye movements, blinks, and muscle activity were rejected, (c) data were then separated into one‐second epochs, and (d) the baseline was defined as the mean amplitude in the presegment data of 200 ms.

**Table 1:** Demographic data (means ± *SD*) of ASD and TD group

	ASD	TD	*p*
*N*	13	15	
Age range, years	3.75–7.91	4.44–6.83	
Age, years	5.44 ± 1.68	5.86 ± 0.66	.410
Sex (male/ female)	11/2	15/0	.206
Handedness (right/ left)	12/1	15/0	.464
PPVT	70.58 ± 19.66	98.67 ± 22.62	**.002**
ADI‐R subscale			
Social interaction	20.08 ± 5.71	N/A	N/A
Communication	17.67 ± 3.70	N/A	N/A
Restricted, repetitive, and stereotyped behaviors	6.92 ± 2.39	N/A	N/A
Early development	3.33 ± 1.37	N/A	N/A
ADOS subscale			
Communication	5.67 ± 1.97	N/A	N/A
Social interaction	9.25 ± 1.77	N/A	N/A
Communication and social interaction	14.92 ± 3.15	N/A	N/A
Stereotyped behaviors and restricted interests	1.83 ± 1.19	N/A	N/A
SRS total score	78.92 ± 25.11	N/A	N/A
SRS subscale			
Social perception	4.31 ± 5.70	N/A	N/A
Social cognition	17.69 ± 5.77	N/A	N/A
Social communication	30.08 ± 9.26	N/A	N/A
Social motivation	14.23 ± 6.30	N/A	N/A
Autism behavior pattern	12.62 ± 5.82	N/A	N/A
AQ‐child total score	77.91 ± 24.13	N/A	N/A
AQ‐child subscale			
Social skill	16.73 ± 6.83	N/A	N/A
Attention switching	13.09 ± 3.48	N/A	N/A
Attention to details	14.00 ± 6.16	N/A	N/A
Communication	16.91 ± 6.85	N/A	N/A
Imagination	17.18 ± 4.81	N/A	N/A

Abbreviations: ADI‐R, Autism Diagnostic Interview‐Revised; ADOS, Autism Diagnostic Observation Schedule; AQ‐child, Autism Spectrum Quotient Children's Version; ASD, autism spectrum disorder; N/A, not applicable; PPVT, Peabody Picture Vocabulary Test; *SD*, standard deviation; SRS, Social Responsiveness Scale; TD, typical developing.

Bold values means *p* <.05

### 2.5 EEG power analysis

After fast Fourier transform (FFT), we extracted the power values of Fz, Pz, Oz, and Cz electrodes of four frequency bands from all participants’ all preprocessed data and calculated the average value.

### 2.6 EEG coherence analysis

**Figure 1:**
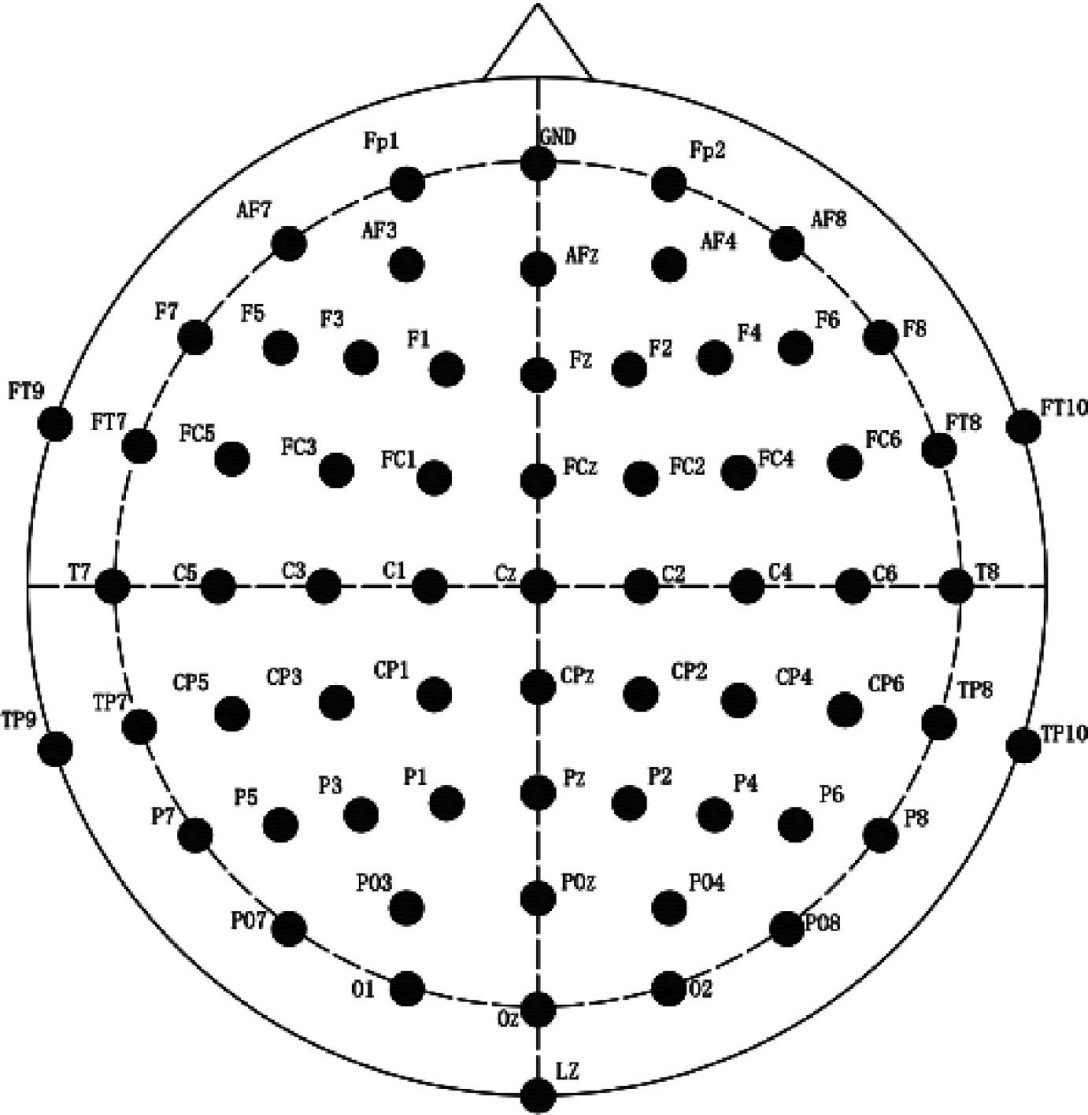
The distribution of 64 electrodes in the brain

Coherence, reflects quantitatively the degree of coincidence of paired EEG signals, was also conducted by the Brain Vision Analyzer software (Brain Products, GmbH). Firstly, FFT converted signal of time domain to frequency domain of EEG data. Then, coherence analysis was computed for eight long‐distance electrode pairs with more than 10–12 cm (F3‐O1, F4‐O2, F7‐F8, F3‐F4, T7‐T8, P7‐P8, C3‐C4, P3‐P4) and eight short‐distance electrode pairs with <10 cm (Fp1‐F3, Fp2‐F4, Fp1‐Fp2, C3‐P3, C4‐P4,T8‐P8, T7‐P7, O1‐O2). The distribution of brain electrodes was shown in Figure 1.

### 2.7 Statistical analysis

The statistical analysis was conducted by using SPSS 19.0. We used Kruskal–Wallis nonparametric statistics to compare the age, handedness, and IQ of the two groups. Then, between‐groups differences within coherence in 16 electrode pairs and power value of four frequency bands were obtained using two‐sample two‐tailed *t* tests. The present study explored whether altered coherence was associated with severity of symptoms in ASD. Pearson correlation analysis was utilized to determine the relationship between the atypical coherence values and the ADI‐R, ADOS, SRS, and AQ scores in ASD group.

## 3 RESULTS

### 3.1 Between‐group differences in EEG power

Using Two‐sample two‐tailed *t* tests, we found that compared to TD, children with ASD showed significantly increased power value in the delta band at Fz compared with TD (*t* = 3.21, *p* = .01; Table 2).

**Table 2:** Group comparison of power values (µV^2^)

Bands	Electrodes	ASD	TD	*t*	*p*
Delta	Fz	0.39 ± 0.26	0.15 ± 0.09	3.21	**.01**
	Pz	0.19 ± 0.19	0.09 ± 0.05	1.82	.09
	Oz	0.14 ± 0.19	0.06 ± 0.05	1.60	.12
	Cz	0.35 ± 0.42	0.17 ± 0.12	1.56	.13
Theta	Fz	0.10 ± 0.12	0.05 ± 0.02	1.72	.10
	Pz	0.08 ± 0.06	0.05 ± 0.03	1.55	.13
	Oz	0.04 ± 0.03	0.03 ± 0.02	1.10	.29
	Cz	0.09 ± 0.10	0.07 ± 0.04	0.87	.39
Alpha	Fz	0.02 ± 0.02	0.03 ± 0.02	−0.58	.57
	Pz	0.05 ± 0.08	0.05 ± 0.03	−0.04	.97
	Oz	0.04 ± 0.05	0.03 ± 0.03	0.62	.54
	Cz	0.03 ± 0.02	0.03 ± 0.03	−0.44	.66
Beta	Fz	0.006 ± 0.003	0.005 ± 0.002	1.15	.26
	Pz	0.005 ± 0.003	0.005 ± 0.003	−0.24	.81
	Oz	0.005 ± 0.003	0.005 ± 0.003	−0.61	.55
	Cz	0.004 ± 0.003	0.004 ± 0.002	0.03	.98

Abbreviations: ASD, autism spectrum disorder; TD, typical developing.

Bold values means *p* <.05

### 3.2 Between‐group differences in short‐/long‐distance coherence

**Figure 2:**
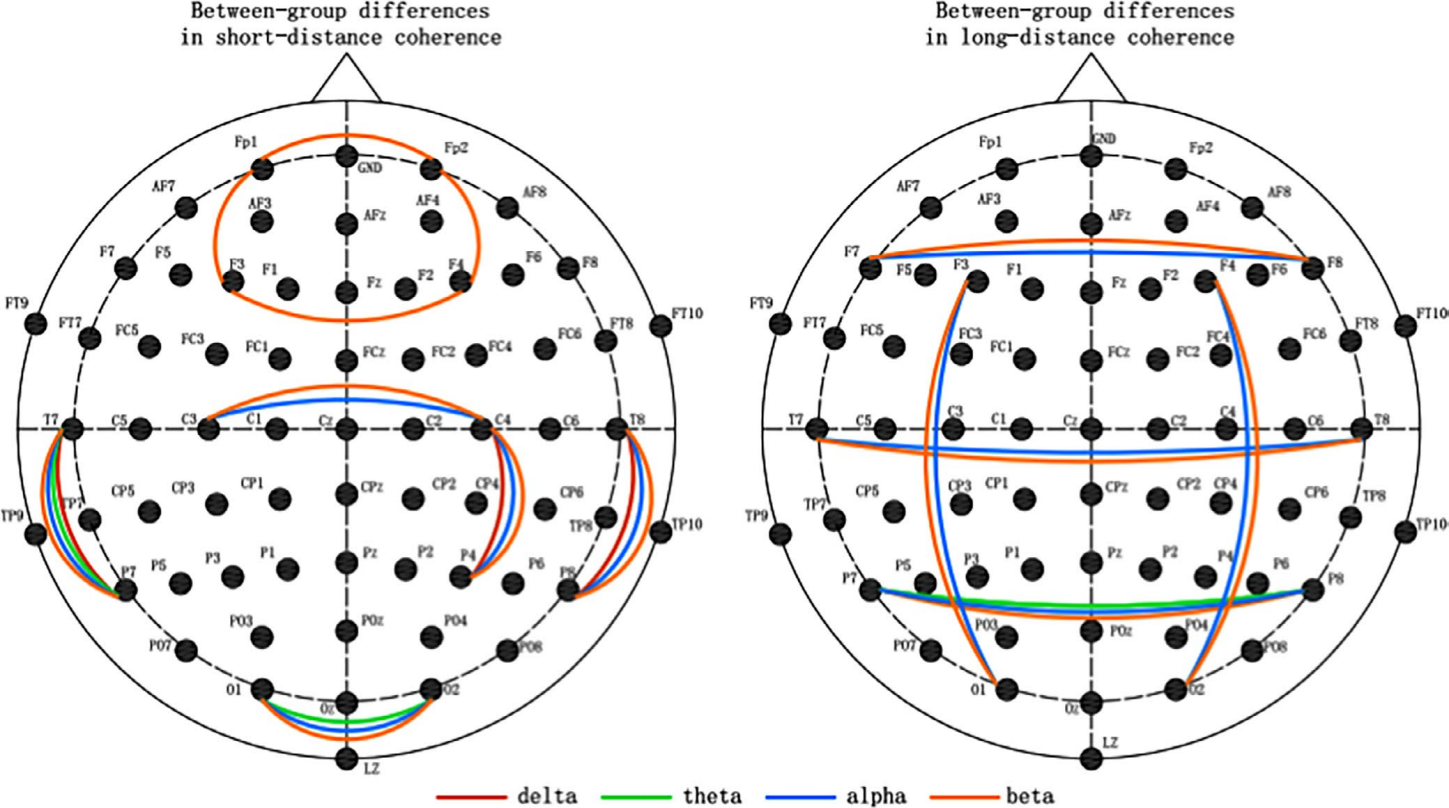
Coherence showing between‐group significant differences in short‐distance and long‐distance connectivity (*p* < .05). The red color lines denotes that ASD group have significantly increased coherence in delta band; the green color lines denotes the significantly increased coherence in ASD group in theta band; the blue color lines denotes the significantly increased coherence in ASD group in alpha band; the orange color lines denotes the significantly increased coherence in ASD group in beta band

In the comparison of short‐distance coherence, the coherence of bilateral temporal–parietal regions T7‐P7 and T8‐P8 electrode pairs was higher in ASD group in all frequency bands. In the comparison of coherence in occipital region, children with ASD showed increased coherence of O1‐O2 electrode pair in theta, alpha, and beta bands. And ASD group had an increased coherence of right central–parietal C4‐P4 electrode pair in delta, alpha, and beta bands. In the comparison of coherence in prefrontal region, Fp1‐Fp2, Fp1‐F3, F3‐F4, and Fp2‐F4 electrode pairs had increased coherence in beta band (Figure 2, Table 3).

**Table 3:** Coherence values of group differences in short‐/long‐distances electrode pairs (mean ± *SD*)

Band	Electrode pairs	ASD	TD	*p*
Short distances				
Delta	T7‐P7	0.09 ± 0.11	0.03 ± 0.02	.046
	T8‐P8	0.06 ± 0.08	0.02 ± 0.02	.028
	C4‐P4	0.2 ± 0.16	0.08 ± 0.05	.025
Theta	T7‐P7	0.12 ± 0.11	0.04 ± 0.03	.019
	O1‐O2	0.27 ± 0.16	0.13 ± 0.07	.015
Alpha	T7‐P7	0.15 ± 0.09	0.05 ± 0.06	.004
	T8‐P8	0.1 ± 0.08	0.03 ± 0.03	.015
	C4‐P4	0.18 ± 0.12	0.09 ± 0.04	.015
	C3‐C4	0.17 ± 0.15	0.04 ± 0.02	.043
	O1‐O2	0.25 ± 0.15	0.13 ± 0.07	.024
Beta	FP‐1F3	0.22 ± 0.13	0.07 ± 0.05	.001
	T7‐P7	0.16 ± 0.11	0.02 ± 0.01	.001
	FP2‐F4	0.26 ± 0.19	0.06 ± 0.07	.004
	T8‐P8	0.09 ± 0.08	0.02 ± 0.02	.018
	C4‐P4	0.22 ± 0.1	0.14 ± 0.07	.013
	FP1‐FP2	0.27 ± 0.14	0.11 ± 0.1	.001
	F3‐F4	0.17 ± 0.09	0.08 ± 0.04	.005
	C3‐C4	0.22 ± 0.12	0.08 ± 0.05	.010
	O1‐O2	0.32 ± 0.15	0.11 ± 0.07	.001
Long distances				
Theta	P7‐P8	0.06 ± 0.06	0.02 ± 0.01	.024
Alpha	F3‐O1	0.06 ± 0.07	0.01 ± 0	.043
	F4‐O2	0.05 ± 0.05	0.01 ± 0.01	.041
	F7‐F8	0.11 ± 0.1	0.05 ± 0.04	.045
	T7‐T8	0.07 ± 0.08	0.01 ± 0.01	.015
	P7‐P8	0.06 ± 0.05	0.03 ± 0.03	.017
Beta	F3‐O1	0.08 ± 0.08	0.01 ± 0.01	.006
	F4‐O2	0.06 ± 0.06	0.01 ± 0.01	.008
	F7‐F8	0.12 ± 0.08	0.04 ± 0.02	.003
	T7‐T8	0.07 ± 0.06	0.02 ± 0.02	.022
	P7‐P8	0.07 ± 0.07	0.02 ± 0.01	.027

Abbreviations: ASD, autism spectrum disorder; TD, typical developing.

In the comparison of long‐distance coherence, ASD group showed increased coherence in alpha and beta bands, including F7‐F8 electrode pair in bilateral frontal regions, T7‐T8 electrode pair in bilateral temporal region, P7‐P8 electrode pair in bilateral parietal region, and F3‐O1, F4‐O2 electrode pairs in bilateral frontal–occipital region. In addition, the coherence of T7‐T8 electrode pair in ASD group was higher in theta band (Table 3).

### 3.3 Relationship with ASD symptoms

In our study, we explore the relationship between aberrant coherence at different frequency bands and the AQ and ADI‐R scores of the children with ASD. The results showed significant positive correlations between the increased coherence in O1‐O2 electrode pair and total scores of AQ in theta, alpha, and beta bands. In beta band, the coherence of T8‐P8, F3‐O1, and F7‐F8 were positively correlated with the detail attention scores of AQ. And ASD group had positive correlation between the coherence of O1‐O2 and F7‐F8 and communication skills of AQ. In addition, the coherence of O1‐O2 in theta and alpha bands and F3‐F4 in beta band was positively correlated with the early developmental abnormality of ADI‐R (Tables 4 and 5).

**Table 4:** Correlations between coherence and AQ scale scores in different frequency bands

Band	Electrode pair	Total scores		Social skill		Attention switch		Attention to details		Communication		Imagination	
		*r*	*p*	*r*	*p*	*r*	*p*	*r*	*p*	*r*	*p*	*r*	*p*
Alpha	O1‐O2	.774	**.014**	.270	.482	.103	.792	.369	.329	.660	.053	.544	.130
Beta	O1‐O2	.864	**.003**	.102	.794	.317	.406	.426	.252	.672	**.047**	.619	.076
	T8‐P8	.456	.159	.218	.521	.288	.39	.738	**.010**	.587	.058	.560	.073
	F3‐O1	.318	.340	.284	.398	.123	.719	.635	**.036**	.531	.093	.449	.166
	F7‐F8	.420	.227	.602	.066	.403	.249	.702	**.023**	.667	**.035**	.633	.050
Theta	O1‐O2	.689	**.040**	.185	.633	−.109	.780	.172	.657	.464	.208	.360	.342

Bold values means *p* <.05

**Table 5:** Correlations between coherence and ADI‐R scale scores in different frequency bands

Band	Electrode pair	Social interaction		Communication		Restricted behaviors		Early development	
		*r*	*p*	*r*	*p*	*r*	*p*	*r*	*p*
Alpha	O1‐O2	.549	.100	.412	.237	.044	.903	.814	**.004**
Beta	O1‐O2	.310	.384	.140	.700	−.114	.755	.536	.111
Theta	O1‐O2	.461	.180	.284	.427	.027	.940	.678	**.031**
Delta	O1‐O2	.420	.227	.141	.697	−.046	.900	.535	.111
Alpha	F3‐F4	.294	.354	.020	.952	−.535	.073	.392	.207
Beta	F3‐F4	.152	.637	.462	.130	−.542	.069	.624	**.030**
Theta	F3‐F4	.229	.475	.261	.412	−.399	.199	.308	.331
Delta	F3‐F4	.022	.947	.160	.620	−.337	.283	.112	.728

Bold values means *p* <.05

## 4 DISCUSSION

The brain has an extremely complex neural network structure. The functional integration and functional separation of neurons in different brain regions are the neural basis of brain information processing (Tononi, Sporns, & Edelman, 1994). In power analysis, children with ASD showed significantly increased power value in frontal region in the delta band. Our present study represented abnormal connectivity patterns in children with ASD. The coherence analysis of four frequency bands (delta, theta, alpha, and beta) found that the coherence of short‐distance connection and long‐distance connection in children with ASD was higher, compared with healthy children. Furthermore, correlation analysis shows that the abnormal EEG coherence was associated with clinical scale scores, further demonstrating the robustness of our findings.

Delta frequency power trajectories consistently distinguish infants with ASD diagnoses from others (Gabard‐Durnam et al., 2019). In present study, children with ASD showed significantly increased delta power value over frontal region. In support, a trend toward elevated delta power in children with ASD diagnosis at frontal scalp regions was indicated (Shephard et al., 2018). Therefore, the increased delta power value in the frontal region is a typical manifestation in children with ASD, which can be used as one of the neuroelectrophysiological markers for ASD.

The EEG coherence signals in resting state contain complex physiological information, which reflects the integration and processing of information by the brain in resting state and reflects the synchronization of the global brain neuron activity (Lord & Opacka‐Juffry, 2016). In the theory of nerve pruning, as the age of normal children grows, the myelin sheath of neurons matures and is further pruned and modified (Chugani, Phelps, & Mazziotta, 1987; Huttenlocher, De Courten, Garey, & Hendrik, 1982). The functional connections between the local or adjacent brain regions decreased, while the functional connections between remote brain regions increased (Fair et al., 2007). We found that children with ASD had excessive short‐distance connections in the left and right temporal–parietal regions, the right central–parietal regions, and the occipital regions. We speculate that enhanced short‐distance connections in children with ASD may due to over‐groomed nerves synaptic pruning. Additionally, synaptic abnormal theory hypothesizes that the enhanced functional integration in local brain regions may be contributed to the aberrant balance of excitation and inhibition in local neural circuits (Testa‐Silva et al., 2012; Tuchman & Cuccaro, 2011; Yizhar et al., 2011), which is caused by either increased synaptic excitation or decreased synaptic inhibition (Yizhar et al., 2011). This aberrant balance has also been supported by blood biochemical studies in children with ASD (Cellot & Cherubini, 2014). Besides, we found that the coherence of children with ASD in the prefrontal region was significantly increased. Carper points out that the increased frontal lobe volume was obvious in the early brain development of autistic children (Carper, Moses, Tigue, & Courchesne, 2002). This transitional growth may lead to excessive connections in the frontal lobe (Coben & Myers, 2008; Rinaldi, Perrodin, & Markram, 2008), interfering with normal growth and development trajectories in ASD.

On the other hand, we found the excessive long‐distance connections in frontal region, temporal region, parietal region, and frontal–occipital region, which was inconsistent with many studies about the reduced long‐distance connection (Khan et al., 2013; Kikuchi et al., 2015). Aberrant brain long‐distance connections were reported in autistic children (Coben, Clarke, et al., 2008; Duffy & Als, 2012), adolescents (Lajiness‐O'Neill et al., 2014), and adults (Leveille et al., 2010; Mathewson et al., 2012; Saunders, Kirk, & Waldie, 2016). However, there is no consistent conclusion about brain long‐distance connection patterns in different autistic age groups. A recent fMRI study provided an imaging evidence for our findings (Supekar et al., 2013). It pointed out that the hyperconnectivity was observed at the whole‐brain and subsystems level, across long‐ and short‐range connections in a large sample with 110 children with ASD, and children with more severe impairment in the social domain exhibited increased functional connectivity. Therefore, the aberrant short‐/long‐distance connectivity may be associated with pathogenic mechanisms in children with ASD. Long‐distance connectivity is a higher‐level brain activity, which rapidly integrates information in different brain areas into a consistent behavioral or cognitive state. Beta signal is mainly observed in brain frontal and central areas, and related to positive thinking and concentration of mind (Pfurtscheller & Lopes da Silva, 1999). Our result showed that ASD had increased long‐distance coherence in the frontal and central regions in beta band; this might suggest a dysfunctional connectivity pattern of some brain regions in children with ASD. In resting state, information integration and processing need multiple brain regions to cooperate with each other. According to neural compensatory mechanism, the increased long‐distance coherence may represent compensatory processes or reduced neural pruning (Duffy & Als, 2012). This may constitute a compensatory attempt of the autistic brain to form atypical, spatially disparate, cortical networks in an attempt to replace function normally subserved by assumed‐to‐be deficient more localized networks. This compensatory mechanism may be the cause of increased long‐distance coherence in children with ASD.

Correlation analysis shows that increased short‐distance connection in the occipital area (O1‐O2) was positively correlated with symptom severity, which is supported by imaging evidence (Nair et al., 2018). The occipital striate area is the central visual cortex as well as the end of the nerve fibers that transmit information from the retina. The pattern of excessive connection of occipital striate area may be the characteristic change in children with ASD and be correlated with symptom severity. Furthermore, Carson et al. (2014) found that the abnormal coherence in alpha band was related to priority attention to details in children with ASD. In present study, we found that there was a positive correlation between left frontal–occipital, frontal region, and right temporal–parietal region and attention to details of AQ in beta band. Consequently, the abnormal connection pattern of children with ASD may be a neurophysiological basis for their clinical symptoms even though the results of the previous studies were not consistent.

The prefrontal cortex is the core area of the "social brain" (Dunbar, 1998). The activation of prefrontal cortex is associated with cognitive control tasks, and frontal lobe dysfunction is related to social, emotional, and cognitive impairment in ASD (Rajak et al., 2018). In present study, the hyperconnections in prefrontal region, bilateral temporal–parietal, were not related to social and cognitive ability in children with ASD. Children with ASD showed excessive connections in the prefrontal lobe, which means more neurons were activated during social information processing, and further result in cognitive, social, and emotional dysfunction. The temporal lobe is responsible for processing auditory information. Our results showed that children with ASD had increased local connections in bilateral temporal–parietal regions (T7‐P7, T8‐P8). The spatial location of P7 and P8 electrodes is related to Wernicke's region. Wernicke's region is the center of language understanding. It might indicate that children with ASD have difficulty in language comprehension when they deal with auditory information.

### 4.1 Limitation

Our study had some limitations. First, our sample in this study was small. Hence, we did not group the subjects according to their cognitive ability. It was worth exploring whether the research results are generalized to large sample after grouping the children according to their intelligence level. Second, in this study, only a small number of female subjects were included in the ASD group. It is undeniable that sexual dimorphism in autism is an important factor that should be considered, although there is no difference between the two groups on sex. In future research, we will further explore whether the EEG coherence of children with ASD of different genders will show different characteristics. Third, we use the analysis software of BP company in Germany for data processing in this study. In the future research, we will use more sophisticated analysis software, such as MATLAB software package for more in‐depth analysis.

## CONFLICT OF INTEREST

The authors declare that the research was conducted in the absence of any commercial or financial relationships that could be construed as a potential conflict of interest.

## AUTHOR CONTRIBUTION

Jia Wang conceived of the study, performed the data acquisition, and drafted and revised the manuscript. Xiaomin Wang was involved in the writing and revision of the manuscript, and contributed to the acquisition of clinical data. Xuelai Wang and Lei Chen was responsible for the analysis and interpretation of EEG data and helped to draft the manuscript. Huiying Zhang, Yong Zhou, and Yutong Li revised it and made instructive recommendations. Lijie Wu conceived of the study and helped to receive and revise the manuscript. All authors read and approved the final manuscript.
